# HEALTH-RELATED QUALITY OF LIFE IN OLDER ADULTS AFTER HURRICANE IDA

**DOI:** 10.1093/geroni/igae098.0861

**Published:** 2024-12-31

**Authors:** Piper Bordes, Katie Cherry

**Affiliations:** Louisiana State University, Baton Rouge, Louisiana, United States; Louisiana State University, Baton Rouge, Louisiana, United States

## Abstract

Hurricane Ida made landfall in Grand Isle, Louisiana on August 29, 2021, the sixteenth anniversary of the 2005 Hurricane Katrina, which devastated the US Gulf Coast. Hurricane Ida claimed 87 lives in the US and caused an estimated 75 billion dollars in damages. In the present study, we examined post-disaster health and well-being in directly and indirectly affected younger and older adults. A total of 128 participants were sampled from three parishes (counties) where the storm devastation was more severe and two parishes farther inland with less damage. All were tested in person or virtually via the Zoom platform in a single session. They responded to several individual difference measures, a structured storm questionnaire and the SF-36 Health-Related Quality of Life Survey. Results indicated the physical health composite score was poorer for older adults, as expected (p <.001). In contrast, mental health scores were higher for the older adults, although age interacted with exposure status where the size of the age difference was reduced for those who were directly impacted by Ida (p <.01). Implications of these data for understanding older adults’ psychological health after decades of severe weather events are discussed.

